# Development and Validation of a Novel Risk Calculator to Predict Sub-optimal HIV Outcomes Among Pregnant and Postpartum Women with HIV in Kenya

**DOI:** 10.1007/s10461-025-04814-8

**Published:** 2025-07-10

**Authors:** Kevin Owuor, Janet M. Turan, Jeff M. Szychowski, Maricianah Onono, Linet Ongeri, Laura K. Beres, Anna Helova, Emmah Ouma, Mercelline Onyando, Rena C. Patel, Patrick Oyaro, Lisa L. Abuogi, Karen Hampanda

**Affiliations:** 1 Department of Biostatistics, School of Public Health, The University of Alabama at Birmingham, Birmingham, AL USA; 2 Sparkman Center for Global Health, School of Public Health, The University of Alabama at Birmingham, Birmingham, AL USA; 3 Department of Health Policy and Organization, School of Public Health, The University of Alabama at Birmingham, Birmingham, AL USA; 4 Centre for Microbiology Research, Kenya Medical Research Institute, Nairobi, Kenya; 5 Centre for Clinical Research, Kenya Medical Research Institute, Nairobi, Kenya; 6 Department of Pediatrics, School of Medicine, University of Colorado Denver, Aurora, CO USA; 7 Department of International Health, Johns Hopkins Bloomberg School of Public Health, Baltimore, MD USA; 8 Department of Infectious Diseases, School of Medicine, The University of Alabama at Birmingham, Birmingham, AL USA; 9 Department of Obstetrics and Gynecology, School of Medicine, University of Colorado Anschutz Medical Campus, Aurora, CO USA; 10 Health Innovations Kenya (HIK), Kisumu, Kenya

**Keywords:** HIV, Pregnant, Postpartum, Risk, Virologic Failure, Care Disengagement

## Abstract

No tool currently exists to predict the cumulative risk of suboptimal clinical outcomes among pregnant and postpartum women with HIV (PPWH). This study sought to develop and validate a parsimonious risk calculator capable of predicting disengagement from care and HIV treatment failure among PPWH. We created the risk calculator using data from 1,331 PPWH from Southwestern Kenya (Homabay, Migori, and Kisumu Counties) in the Mother Infant Visit Adherence and Treatment Engagement Trial. Least absolute shrinkage and selection operator logistic regression retained the most predictive variables from 16 candidate factors to estimate the probability of treatment failure or disengagement from care. Three risk quintiles were calculated. We assessed external validation with an independent dataset (Opt4Mamas; *N* = 820). Cross-validated area under the curve of receiver operating characteristic (AUROC) and calibration measures assessed model performance. Two unique risk calculators were created - one for PPWH with known HIV diagnosis prior to pregnancy and one for PPWH with new HIV diagnoses. The combined outcome of care disengagement or treatment failure occurred in 43% of PPWH with known diagnosis and 40% with new diagnosis in the development dataset; and 37% with known diagnosis and 13% with new diagnosis in the validation dataset. The calculators included demographic (age, parity, marital status), clinical (virological failure, missed visits, regimen line, gestation age), and psychosocial variables (intimate partner violence, stigma, depression, partner support, disclosure, food insecurity). The model for PPWH with known diagnosis demonstrated better calibration and discrimination (AUROC 0.843 [95% CI 0.805, 0.866]) than the model for PPWH with a new HIV diagnosis (AUROC 0.463 [95% CI 0.347, 0.597]). Mean predicted risk probabilities among PPWH with known HIV diagnosis were: low (6%), moderate (56%), and high (70%). Mean predicted risk probabilities among those with a new HIV diagnosis were: low (31%), moderate (48%), and high (65%). The novel risk calculator for PPWH with a known HIV diagnosis has the potential to identify those who are at risk of sub-optimal HIV treatment and care outcomes for targeted interventions to prevent treatment failure and loss to follow-up.

**Supplementary Information:**

The online version contains supplementary material available at 10.1007/s10461-025-04814-8.

## Introduction

Pregnant and postpartum women with HIV (PPWH) experience varied success with HIV treatment and maternal and child health outcomes [1]. The proportion of PPWH accessing antiretroviral therapy (ART) has steadily increased, with the most recent estimates (2023) indicating that 84% of PPWH globally have access to ART [1]. There has been a reduction in mother-to-child transmission (MTCT) globally from 22% in 2010 to 10% in 2023; however, the target of elimination of MTCT has not been achieved [2, 3]. Poor retention of women on antiretroviral therapy (ART) during pregnancy and breastfeeding is a major contributing factor for MTCT (5), with a quarter of African PPWH lost to follow-up within six months of starting ART [4]. In Eastern Africa, 40% of new pediatric infections are estimated to be attributable to PPWH stopping ART [5]. Additionally, maintaining high adherence to ART and continuous viral suppression during the postpartum period remains challenging [6–8].

Prediction tools using a risk score have successfully been implemented in both high and low-resource settings to identify patients with various health conditions at risk of specific outcomes, including HIV [9–13]. Prediction tools have been used to detect patients who are at high risk of acquiring HIV [14–19], disengaging from care after HIV diagnosis [20] and to identify non-pregnant/postpartum patients with HIV who are likely to have treatment failure due to a combination of clinical (prior treatment failure, reported non-adherence, missed clinic visits) [21] and psychosocial (homelessness, mental health conditions, violence/trauma, stigma) factors [17, 22]. A study from western Kenya with pregnant/postpartum women without HIV reported the utility of a risk stratification tool; capturing male partner HIV status, number of sexual partners, and diagnosis of sexually transmitted infections (STIs); for predicting acquisition of HIV [23].

Despite numerous known risk factors for poor treatment outcomes and loss to follow up (LTFU), including intimate partner violence (IPV), stigma, depression, young age, new HIV diagnosis [4, 24–39], no tool currently exists to predict a PPWH’s cumulative risk of disengagement from care or treatment failure. There is a pressing need for such a tool, given the differential success of PPWH and limited resources available to support maternal and child health goals and eliminate MTCT. A validated prediction tool has the potential to help researchers and clinicians identify PPWH at-risk of disengagement from care and treatment failure to intervene before negative outcomes, such as MTCT, occur [20, 40–42]. The objective of this study was to develop and validate a novel multivariable prediction tool (i.e., “risk calculator”) for the combined outcome of disengagement from care or elevated viral load among PPWH using risk prioritization scores (high, moderate, low) based on socio-demographic, clinical, and psychosocial indicators known to be associated with sub-optimal outcomes among PPWH.

## Methods

### Sources of Data

#### Development Dataset

A longitudinal quantitative dataset from the Mother-Infant Visit Adherence and Treatment Engagement (MOTIVATE) randomized trial with 1,331 PPWH (18–45 years) from 24 government-run clinics in Kisumu, Migori, and Homa Bay Counties and associated sub-studies was used to develop the risk calculator (i.e., derivation dataset) [43]. The study was conducted among PPWH recruited between December 2015 and August 2017 and followed up to March 2019. Data from the parent MOTIVATE study included demographics, retention in care (HIV care visit within 90 days at 12 months postpartum), and routinely collected viral load. The sub-studies included a detailed follow-up psychosocial survey at 12 months postpartum with a sub-sample of MOTIVATE participants (*n* = 200), and a survey among PPWH who were lost-to-follow-up or discontinued from the main study and who could subsequently be traced (*n* = 79).

#### Validation Dataset

The Opt4Mamas study (*N* = 767 PPWH) was used as the validation dataset. Opt4Mamas was a pre- and post-implementation cohort study of a point-of-care (POC) viral load testing intervention among PPWH (18–45 years) in five government clinics in Kisumu, Kenya [44]. Participants were enrolled starting in February 2019 and followed through December 2021. Viral load and extensive psychosocial data were collected during pregnancy (baseline), delivery, and six months postpartum. The variables used for model validation were similar to those used for model development from the MOTIVATE trial [43].

### Setting

Homabay, Migori, and Kisumu Counties are in Western Kenya and all border Lake Victoria. Homabay County has the highest HIV prevalence rate in Kenya, estimated at 19.6% followed by Kisumu (17.5%) and Migori (13.3%) Counties according to the 2018 Kenya Population-based HIV Impact Assessment (KENPHIA) [45]. Homabay, Kisumu, and Migori Counties have a combined annual estimate of over 17,000 pregnant women with HIV, with high perinatal HIV transmission rates of 7.2–8.7% [46, 47]. During the study periods, all Kenya Ministry of Health (MoH) facilities provided ART free of charge for all PPWH and routine viral load (VL) testing every six months (initial VL after 6 months of ART for women with new HIV diagnoses and upon pregnancy for those already on ART) through breastfeeding per MoH guidelines. At the time of the MOTIVATE study, HIV viral suppression was considered < 1,000 copies/ml; at the time of the OPT4MAMAS study and secondary data analysis for the present study, HIV viral suppression was considered < 400 copies/ml per MoH guidelines.

### Outcome

The outcome of interest for the risk prediction tool was defined as a combined binary outcome of disengagement from care (missed HIV visit ≥30 days) or elevated viral load (≥400 copies/ml) among PPWH at 12 months postpartum.

### Predictors

A total of 16 demographic, clinical, and psychosocial candidate predictors were assessed for potential inclusion in the risk calculator based on our team’s prior research [43, 44, 48–50] and existing literature (Table 1) [4, 24–39].

**Table 1:** Risk factor variables used in model development

Variables	Known HIV diagnosis		New HIV diagnosis	
Demographic predictors	Assessed	Included in Final Model	Assessed	Included in Final Model
Age (Young age [< 26 years] vs. older age [≥26 years])	✓	✓	✓	✓
Parity (Nulliparous vs. parous)	✓		✓	✓
Marital status (Not married vs. Married)	✓	✓	✓	✓
Clinical predictors				
Baseline viral load category (< 1000 copies, ≥1000 copies/ml, or Unknown)	✓	✓		
History of viral failure (≥400 copies/ml) after baseline and before 12 months postpartum (Yes vs. No)	✓	✓		
Baseline Adherence (Good, Poor, or Unknown)	✓	✓		
History of missed visit - Past missed clinic visits of > 14 days before 12 months postpartum (Yes vs. No)	✓	✓		
Antiretroviral Therapy Regimen (Second- or third-line regimen vs. First-line)	✓	✓		
Late gestational age at first ANC (> 26 weeks at first ANC visit vs. <=26 weeks)	✓			
Psychosocial predictors				
Depression symptoms on Patient Health Questionnaire PHQ-9 (Yes [≥10] vs. No [< 10]) [46, 47]	✓	✓	✓	
Internalized HIV-related stigma - Endorsed stigma on internalized stigma scale (Yes [any of 6 items > 1] vs. No [all items = 1) [51]	✓	✓	✓	✓
Anticipated HIV-related stigma - Endorsed stigma on anticipated stigma scale (Yes [any of 5 items > 2] vs. No [all items ≤ 2) [48, 52]	✓	✓	✓	✓
Intimate partner violence (Exposure to physical, sexual, or emotional IPV in the past 12 months vs. No)	✓	✓	✓	✓
Low male partner support (Low perceived levels of male partner support from measures of affective support [< 4] vs. High levels [≥4]) [53, 54]	✓	✓	✓	
Low male partner involvement in PMTCT (Low score [> 4] on the male partner involvement scale vs. High [≤ 4]) [49]	✓		✓	
Non-disclosure of HIV status to male partner (Yes vs. No)	✓	✓	✓	
Food insecurity (Food insecure [Mildly, Moderately, or Severely] as per Household Food Insecurity Access Scale vs. Food secure) [55].	✓	✓	✓	✓

PMTCT– Prevention of mother-to-child transmission of HIV

### Sample Size and Power Calculation

The derivation dataset (*N* = 1331) was adequate (≥880 participants needed) to ensure that the developed prediction model using a binary outcome (the combined outcome of a missed visit (≥30 days) or treatment failure (elevated VL≥400 copies/ml) would have a small mean absolute prediction error (MAPE) when applied in other targeted individuals, based on a set MAPE of 0.05 (recommended largest value) with an anticipated outcome of 20% or more with missed visit or treatment failure (based on MOTIVATE outcome data) and up to 12 candidate predictor parameters [51]. Our validation dataset (*N* = 767) was adequate (≥650 participants needed) to ensure small expected optimism in the apparent R^2^_Nagelkerke_ = (R^2^_cs(cox snell)_/max(R^2^_cs_); anticipated R^2^_cs_≥ 0.2; max(R^2^_cs_) = 0.33; delta *≤* 0.05) of the logistic regression model [52].

### Missing Data

Both the development and validation datasets had missing data that was handled using Multivariate Imputation by Chained Equations (MICE). The development dataset had many missing values due to limited psychosocial data in the main dataset; these were collected via two surveys administered to a subsample of 8% of participants in MOTIVATE from the LTFU sub-study (*n* = 78) and MOTIVATE Follow-up sub-study (*n* = 198). These will be referred to as the ‘LFTU sub-study’ and the ‘Follow-up sub-study’, respectively. Missing data patterns were examined using Little’s test to assess whether the data were missing completely at random. Little’s test for covariate-dependent missingness (CDM) assumption was used to infer whether the data were likely missing at random [53]. MICE was conducted with 193 and 20 imputation iterations based on the fraction of missing data for development and validation data, respectively [54–56].

### Statistical Analysis Methods

Separate models were built and assessed for PPWH with a known HIV diagnosis prior to the pregnancy and those with a new HIV diagnosis, because PPWH with a new HIV diagnosis did not have clinically relevant historical data (e.g., history of elevated viral load). Analytical methods were based on previous studies that have successfully developed predictive tools [17, 22, 57] and TRIPOD (Transparent Reporting of a multivariable prediction model for Individual Prognosis or Diagnosis) criteria [58]. Descriptive analyses of participant characteristics were conducted. Bivariate associations of the outcome and predictors were assessed using Chi-square tests. A least absolute shrinkage and selection operator (Lasso) penalized logistic regression procedure was used to retain the most predictive variables in model development. The Lagrangian form of the Lasso formula is shown below [59].

$$\:{\widehat{\beta\:}}^{lasso}=\genfrac{}{}{0pt}{}{arg\:min}{\beta\:}\left\{\frac{1}{2}\sum\:_{i=1}^{N}{({y}_{i}-{\beta\:}_{0}-\sum\:_{j=1}^{p}{x}_{ij}{\beta\:}_{j})}^{2}+\:\lambda\:\sum\:_{j=1}^{p}\left|{\beta\:}_{j}\right|\right\}$$

Where $\:{\widehat{\beta\:}}^{lasso}$ is a Lasso estimate, $\:{\beta\:}_{0}$ is the intercept, $\:{\beta\:}_{j}\:$are least squares estimates, $\:{y}_{i}$ is virologic failure or disengagement from care outcome, $\:{x}_{ij}$ are predictors, L_1_ Lasso regularization (penalty) term is $\:{\sum\:}_{1}^{p}\left|{\beta\:}_{j}\right|$, and $\:\lambda\:$ is the regularization coefficient.

All predictors were included in the model for the participants with a known HIV diagnosis, while historical clinical predictors were omitted in the model for participants with a new HIV diagnosis. A 10-fold cross-validation method was applied to find the regularization parameter lambda that minimizes the mean squared prediction error (MSPE). The Lasso models were fit to each imputed dataset. Predictors with nonzero coefficients in at least 50% of all models were selected for calculating the predicted risk of the outcome of virologic failure/disengagement in care. Combined linear predictors per participant were calculated for all imputation estimates using Rubin’s rules after confirming the asymptotic normality assumption of the estimates [60]. The linear predictors were used to estimate the probability of treatment failure or disengagement from care from which three quintiles were calculated: Low, Moderate, and High Risk.

Internal validation and performance assessment were done via bootstrapping (1000 resamples) by assessing the cross-validated discrimination and calibration. External validation was done using the separate independent study (Opt4Mamas) dataset. The Brier score as mean squared error (MSE) or MSPE, and concordance statistic (C-statistic; area under the receiver-operating characteristic [AUROC] curve) were used to measure performance and discrimination [61, 62]. Calibration was assessed by plotting predicted probabilities against the observed event proportions.

### Risk Groups

The predicted risk of disengagement from care or treatment failure in either group was defined as Risk_KP_ = exp(LP_KP_) / (1 + exp(LP_KP_)) and Risk_NP_ = exp(LP_NP_) / (1 + exp(LP_NP_)) for participants with known HIV positive diagnoses (KP) and those with new HIV positive diagnoses (NP), respectively. The predicted risks were also categorized as quartiles of prediction scores to reflect high (3rd quartile), moderate (2nd quartile), and low (1st quartile) risks of virologic failure or disengagement from care.

### Ethical Review Statement

The ethical review approval was granted by the Kenya Medical Research Institute Scientific and Ethics Review Unit, the University of Alabama at Birmingham, and the University of Colorado Denver IRBs in the United States. Only de-identified datasets were used for the study, and procedures were conducted in accordance with the Declaration of Helsinki (version 2024).

## Results

### Participant Characteristics

The development dataset included 1,331 participants, as described in Table 2. Most were married (91.5%), were 26 years or older (66.9%), and were known to be living with HIV before the index pregnancy (78.8%). Among all 767 participants in the validation dataset, the majority were married (85.1%), over 26 years old (72.2%), and had known HIV diagnoses at the time of pregnancy (93.7%) (Table 2). There was no significant difference in the primary outcome among PPWH with known diagnoses between the development cohort (43.4%) and validation data (36.9%). The characteristics by outcome and timing of HIV diagnosis in both datasets are shown in Table 3.

**Table 2:** Characteristics of pregnant and postpartum women with HIV (PPWH) in the development and validation datasets

Participant Characteristics	Development Dataset	Validation Dataset
	*N* = 1331	*N* = 767
Demographic Items		
Currently married, n (%)	1218 (91.5)	653 (85.1)
Young age (< 26 years), n (%)	440 (33.1)	213 (27.8)
Parous, n (%)	1211 (91.1)	664 (86.9)
Gestational age < = 26 weeks at first ANC visit, n (%)	792 (59.5)	622 (83.2)
Clinical Items^a^		
Known HIV positive (diagnosed before most recent pregnancy), n (%)	1049 (78.8)	719 (93.7)
First Line ART Regimen, n (%)	1270 (95.4)	546 (80.4)
Baseline Adherence, n (%)		
Fair/Poor	35 (2.6)	2 (0.3)
Good	1063 (79.9)	379 (52.7)
Unknown	233 (17.5)	338 (47.0)
History of missed clinic visit(s) > 14 days, n (%)	818 (63.4)	139 (21.1)
Baseline viral load categories, n (%)		
<1000 copies	999 (75.1)	383 (53.3)
>=1000 copies	129 (9.7)	84 (11.7)
Unknown	203 (15.3)	252 (35.0)
History of viral failure [VL > = 400 copies/ml], n (%)	204 (16.8)	155 (23.2)
Psychosocial Items^b^		
Physical Intimate Partner Violence in past 12 months, n (%)	94 (54.0)	100 (13.0)
Anticipated HIV-related stigma, n (%)	84 (42.4)	177 (23.1)
Internalized HIV-related stigma, n (%)	114 (57.6)	99 (12.9)
Major Depression, n (%)	110 (55.6)	56 (7.3)
Low Male partner support, n (%)	72 (40.4)	66 (8.6)
Non-disclosure of HIV status to male partner, n (%)	33 (16.7)	10 (1.3)
Household Food Insecurity, n (%)	89 (44.9)	245 (31.9)

^a^Only collected on PPWH with a known HIV diagnosis

^b^Psychosocial data was only completed among 198 participants. Imputations were therefore used in the regression models

**Table 3:** Characteristics of pregnant and postpartum women with HIV (PPWH) with and without missed visits or treatment failure

	Development Dataset (*N* = 1331)						Validation Dataset^c^ (*N* = 767)							
	Known HIV Diagnoses *N* = 1049			New HIV Diagnoses *N* = 282			Known HIV Diagnoses *N* = 658				New HIV Diagnoses *N* = 47			
Participant Characteristics	Missed visit or treatment failure			Missed visit or treatment failure			Missed visit or treatment failure				Missed visit or treatment failure			
	No	Yes		No	Yes		No	Yes			No	Yes		
n (%)	594 (56.6)	455 (43.4)	P-value	168 (59.6)	114 (40.4)	P-value	415 (63.1)	243 (36.9)	P-value		41 (87.2)	6 (12.8)	P-value	
Demographic Items														
Not married, n (%)	52 (8.8)	30 (6.6)	0.2	20 (11.9)	11 (9.6)	0.55	60 (14.5)	31 (12.8)	0.54		8 (19.5)	2 (33.3)	0.44	
Young age (< 26 years), n (%)	169 (28.5)	110 (24.2)	0.12	99 (58.9)	62 (54.4)	0.45	110 (26.5)	76 (31.3)	0.19		8 (19.5)	1 (16.7)	0.87	
Nulliparous, n (%)	555 (93.4)	429 (94.3)	0.57	31 (18.7)	22 (19.3)	0.9	47 (11.4)	38 (15.8)	0.10		1 (2.4)	1 (16.7)	0.11	
Gestational age < = 26 weeks at first ANC visit, n (%)	351 (59.1)	280 (61.5)	0.42	94 (56.0)	67 (58.8)	0.64	334 (82.3)	192 (82.1)	0.95		37 (90.2)	5 (83.3)	0.61	
Clinical Items^a^														
First Line ART Regimen, n (%)	559 (94.1)	429 (94.3)	0.9				364 (87.7)	168 (69.1)	< 0.001					
Baseline Adherence, n (%)			0.22						< 0.001					
Fair/Poor	19 (3.2)	12 (2.6)					2 (0.5)	0 (0.0)						
Good	554 (93.3)	417 (91.6)					248 (59.8)	113 (46.5)						
Unknown	21 (3.5)	26 (5.7)					165 (39.8)	130 (53.5)						
Baseline viral load categories, n (%)			0.441						< 0.001					
<1000 copies	484 (81.5)	365 (80.2)					258 (62.2)	115 (47.3)						
>=1000 copies	52 (8.8)	50 (11.0)					3 (0.7)	81 (33.3)						
Unknown	58 (9.8)	40 (8.8)					154 (37.1)	47 (19.3)						
History of missed clinic visit(s) > 14 days, n (%)	224 (39.0)	439 (96.5)	< 0.001				224 (39.0)	439 (96.5)	< 0.001					
History of viral failure [VL > = 400 copies/ml], n (%)	81 (14.3)	84 (18.9)	0.048				0 (0.0)	155 (64.3)	< 0.001					
Psychosocial Items^b^														
Physical Intimate Partner Violence in past 12 months, n (%)	44 (61.1)	33 (47.1)	0.1	9 (50.0)	8 (57.1)	0.69	69 (16.6)	24 (9.9)	0.021		2 (4.9)	1 (16.7)	0.27	
Anticipated HIV-related stigma, n (%)	28 (33.7)	41 (51.9)	0.019	7 (38.9)	8 (44.4)	0.74	114 (27.5)	50 (20.6)	0.05		5 (12.2)	0 (0.0)	0.37	
Internalized HIV-related stigma, n (%)	52 (62.7)	39 (49.4)	0.09	13 (72.2)	10 (55.6)	0.3	64 (15.4)	26 (10.7)	0.09		4 (9.8)	1 (16.7)	0.61	
Major Depression Category, n (%)	46 (55.4)	45 (57.0)	0.84	11 (61.1)	8 (44.4)	0.32	31 (7.5)	16 (6.6)	0.67		3 (7.3)	0 (0.0)	0.49	
Low Male partner support, n (%)	31 (42.5)	26 (36.1)	0.43	9 (50.0)	6 (40.0)	0.57	33 (8.0)	20 (8.2)	0.90		4 (9.8)	1 (16.7)	0.61	
Non-disclosure of HIV status to male partner, n (%)	16 (19.3)	12 (15.2)	0.49	2 (11.1)	3 (16.7)	0.63	1 (0.2)	6 (2.5)	0.012		0 (0)	0 (0)	-	
Household Food Insecurity, n (%)	39 (47.0)	33 (41.8)	0.51	11 (61.1)	6 (33.3)	0.1	150 (36.1)	74 (30.5)	0.14		11 (26.8)	1 (16.7)	0.59	

^a^Only collected on PPWH with a known HIV diagnosis

^b^Psychosocial data was only collected for 198 participants (*n* = 162 for participants with Known HIV Diagnoses and *n* = 36 for those with New HIV Diagnoses). Imputations were used in the regression models

^c^Only 705 out of 767 participants had completed the outcome variable

Notes: Missed visit or treatment failure outcome was defined as disengagement from care (missed HIV visit ≥30 days) or elevated viral load (≥400 copies/ml) among PPWH at any data point in the parent studies up to 12 months postpartum

All p-values were based on Chi-square tests

### Model Development

The set of binary indicator variables used in model development are shown in the models below.

The final Lasso models fit identified with Linear Predictor (LP_KP_) for participants with known HIV diagnosis at the time of pregnancy and Linear Predictor (LP_NP_) for participants with new HIV diagnosis at the time of pregnancy, defined as follows:

LP_KP_ = -2.112 -0.054*I(Age < 26 years = Yes) + 0.047*I(Marital Status = Not Married) + 0.634* *I*(*Baseline VL* ³ *1000 copies/ml)* + 0.637*I(Baseline VL = Unknown) + 0.506*I(History of viral failure [VL ³*400 copies/ml] = Yes)* + 3.222*I(History of missed clinic visit(s) > 14 days = Yes) − 0.494*I(Physical Intimate Partner Violence in past 12 months = Yes) + 0.576*I(Anticipated HIV-related stigma = Yes) − 0.470*I(Internalized HIV-related stigma = Yes) − 0.040* *I(Major Depression = Yes)* + 0.703*I(Low Male partner support = Yes) + 0.245* *I(Non-disclosure of HIV status to male partner = Yes)* + 0.252*I(ART Regimen = Second- or Third-line) − 0.316*I(Baseline Adherence = Good) + 0.189* *I(Baseline Adherence = Unknown)* − 0.248* *I(Household Food Insecurity = Yes) and*

LP_NP_ = 0.218 − 0.137* *I(Age < 26 years = Yes)* − 0.311* *I(Marital Status = Not Married)* + 0.284*I(Nulliparous = Yes) − 0.080*I(Gestation age > 26 weeks = Yes) − 0.148* *I(Physical Intimate Partner Violence in past 12 months = Yes)* + 0.652* *I(Anticipated HIV-related stigma = Yes)* -0.598* *I(Internalized HIV-related stigma = Yes)* + 0.352*I(Low Male Partner Support = Yes) − 0.397* *I(Household Food Insecurity = Yes)*.

The risk of disengagement from care or treatment failure in either group was defined as follows:

Risk_KP_ = exp(LP_KP_) / (1 + exp(LP_KP_)) and Risk_NP_ = exp(LP_NP_) / (1 + exp(LP_NP_)). A sample implementation of the risk tool in REDCap for the Tatua pilot study with REDCap data dictionary (Online Resource 1) and output are provided (Online Resource 2) [63].

### Performance of Risk Models

#### Participants with Known HIV Diagnosis at the time of Pregnancy

**Fig. 1:**
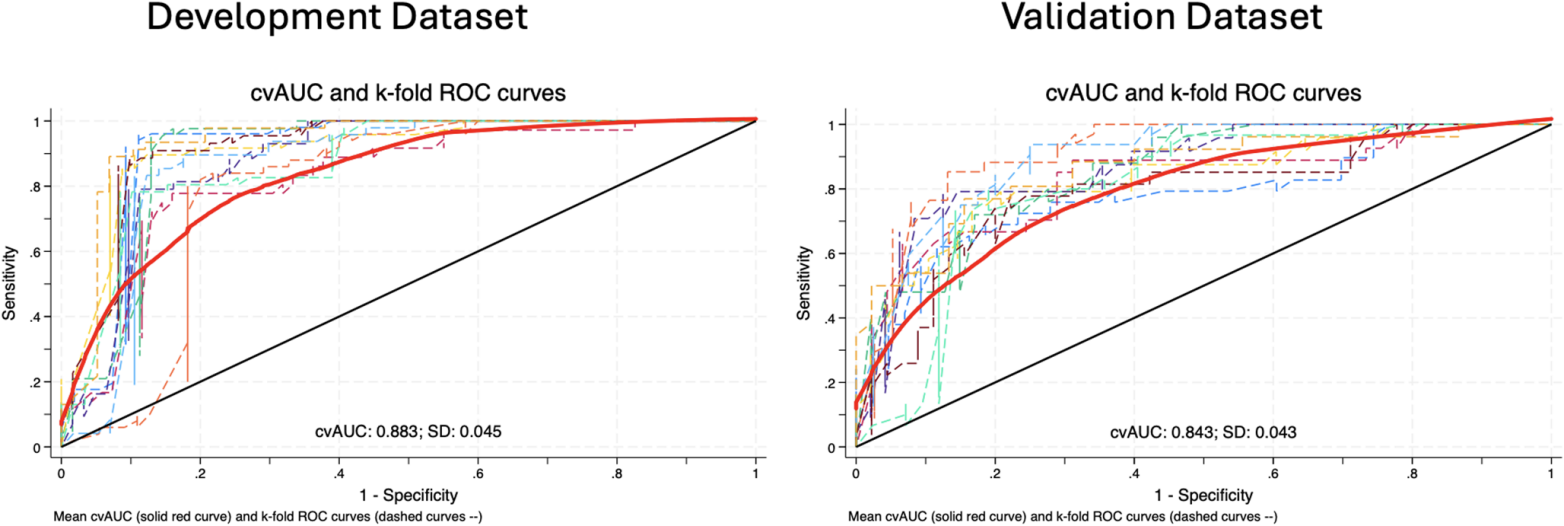
Internal and External Model Discrimination for participants with known HIV diagnosis at the time of pregnancy

The uncalibrated model for participants with known HIV diagnosis overestimated risks (calibration intercept of -0.17). However, the intercept-calibrated model demonstrated good calibration and discrimination (AUROC/C-statistic by bootstrap validation of 0.883 (95% CI 0.847, 0.892) as shown in Table 4; Fig. 1. The externally validated model underestimated risks (calibration intercept of 0.27). The calibrated and externally validated model demonstrated satisfactory calibration and good discrimination (AUROC/C-statistic by bootstrap validation of 0.843 (95% CI 0.805, 0.866)), as indicated in Table 4; Fig. 1.

**Table 4:** Internal and external risk model performance

Model Performance	Cross validated Mean AUCROC (95% CI^a^)	MSE - Mean Squared Error (MSE)	Mean Squared Prediction Error (MSPE)
Internal Validation - Known HIV diagnosis Model^b^	0.883 (0.847, 0.892)	0.150	
Internal Validation - New HIV diagnosis Model ^d^	0.637 (0.631, 0.642)	0.236	
External Validation - Known HIV diagnosis Model ^c^	0.843 (0.805, 0.866)		0.160
External Validation - New HIV diagnosis^e^	0.463 (0.347, 0.597)		0.126

AUCROC– Area Under Curve Receiver Operating Characteristic, Confidence Interval– CI

^a^Bootstrap bias corrected CI

^b^Intercept calibrated model - intercept of -0.17 and coefficient of 1.37 before calibration

^c^Intercept calibrated model - intercept of 0.27 and coefficient of 1.20 before calibration

^d^No calibration done - intercept of 0 and coefficient of 1.01

^e^No calibration done - intercept of 0 and coefficient of 0.06

#### Participants with New HIV Diagnosis at the time of Pregnancy

The uncalibrated model among participants with new HIV diagnosis indicated no tendency to over or underestimate risks (calibration intercept of 0) and not systematically low or high. Despite the good calibration, the model did not demonstrate strong discrimination defined by a C-statistic of 0.8 or more (AUROC/C-statistic by bootstrap validation of 0.637 (95% CI 0.631, 0.642)) as shown in Table 4. Similarly, the externally validated new HIV diagnosis model had a good calibration intercept, but risks were systematically low risk (calibration coefficient of 0.06). Overall, the model demonstrated lower discrimination (AUROC/C-statistic by bootstrap validation of 0.463 (95% CI 0.347, 0.597) as shown in Table 4.

#### Distribution of Predicted Risks of the Outcome by Risk Group

**Fig. 2:**
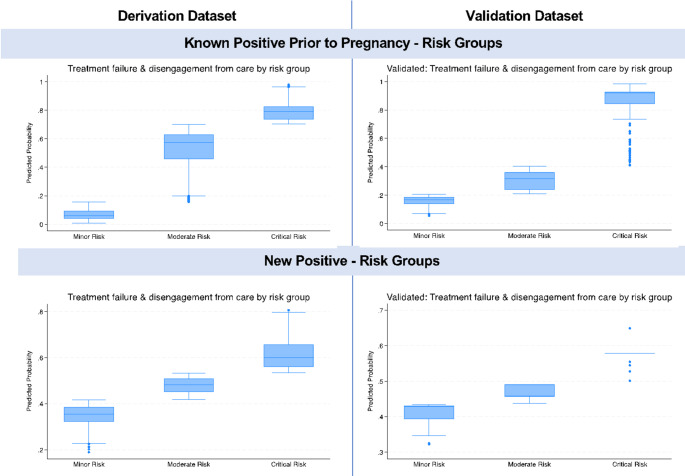
Probability of treatment failure/disengagement from care by risk group in final models

The model-predicted risk groups for the development and external validation (Fig. 2) showed similar distributions in the predicted risks for both participants with known and new HIV diagnoses. The mean predicted risk of the outcome in the low-risk group, moderate-risk group, and high-risk group was 6.1%, 55.8%, and 69.8%, respectively, for PPWH with known HIV diagnoses.

The model-predicted risk groups for the development and external validation (Fig. 2) showed similar distributions in the predicted risks for PPWH with known and new HIV diagnoses. The mean predicted risk of the outcome in the low-risk group, moderate-risk group, and high-risk group was 31.1%, 48.4%, and 64.7%, respectively, for PPWH with new HIV diagnoses.

## Discussion

Using two large longitudinal datasets from southwestern Kenya, this study developed and validated a novel risk calculator capable of predicting treatment failure and disengagement from care among PPWH using risk groups (low, moderate, and high). The findings indicate that the risk calculator can successfully predict PPWH at moderate or high risk of experiencing treatment failure or disengagement from HIV care, especially among those with a known HIV diagnosis prior to pregnancy, which is the majority of pregnant women living with HIV in many Eastern African settings currently. This early identification holds significant potential for both clinical practice and research to allocate resources more effectively and implement targeted interventions for better outcomes among PPWH at greatest risk of treatment failure and disengagement from care [4, 26–38, 43, 64].

The risk calculator exhibited different performance levels depending on the timing of HIV diagnosis of the women [6, 39, 69–71]. The final risk model for PPWH with a known HIV diagnosis outperformed those for PPWH with a new HIV diagnosis, indicating potential to identify those who are at risk of sub-optimal HIV treatment and care outcomes. This disparity suggests that the model benefits from the additional clinical history and data available for women with HIV diagnoses before pregnancy, which likely contributes to more accurate risk predictions. Overall, we expect a varied validation performance of models in different location settings, populations, and over time [65]. Several studies have documented that PPWH with a new HIV diagnosis have a high risk of poor retention and treatment failure [4, 38, 66–68]. The difference could also be due to smaller samples of PPWH with a new HIV diagnosis in the development and validation samples.

The variables included in the final model for PPWH with a known HIV diagnosis were similar to risk factors identified in literature among PPWH or other people living with HIV (PLHIV) [25–30, 33–39, 69, 70]. Similarly, for the model with newly diagnosed PPWH, the variables retained in the final model have been identified in existing literature among PPWH or other PLHIV [4, 30, 39, 69, 70]. Neither of the final models included later gestation age at the first antenatal care visit, parity, or low male partner involvement as key risk factors, even though they have been identified in the literature as potential risk factors of disengagement in care [26, 29, 30, 49]. A limitation of the Lasso model in randomly selecting only one of two or more highly correlated variables could explain this [71].

A major strength of this study is the use of two large, independent longitudinal datasets, which provided a robust basis for model development and validation. One of the key innovations is its ability to integrate and assess risk using multiple domains - clinical, demographic, and psychosocial factors, which strengthens the model’s applicability and relevance to the target population. The tool can stratify risk more comprehensively than models focusing on single or limited factors [23, 40–42]. This approach of including psychosocial factors enables more precise identification of PPWH who need additional support for better outcomes, as reported by Ibrahima et al. (2024) [20]. The utility of such a tool early in the care continuum may be especially beneficial in resource-limited settings, where proactive and efficient use of available resources is crucial. The Tatua study is currently testing screening with the risk calculator within a pilot intervention study in Kenya to identify women who may benefit from a psychosocial support intervention, demonstrating its practical application in ongoing research [63].

However, some limitations should be noted. The datasets we used had some participants exposed to clinical trial interventions and may have introduced some selection bias. The risk calculator model for PPWH who are newly diagnosed with HIV should be used with some caution. The risk calculator models for PPWH newly HIV diagnosed were based on smaller samples and did not perform as well as those with a known diagnosis, highlighting the need for additional predictors for refinement and potentially the inclusion of more development and validation data for this subgroup. Viral suppression thresholds in settings such as Kenya are continually changing with time and more effective ART regimens, which makes a standard viral suppression definition among independent and outcome measures challenging. Missing data in the development dataset was an issue, even though rigorous imputation methods were used to address it. Additionally, while the models have been validated in the context of southwestern Kenya, further external validation is necessary to confirm their broader applicability in different geographic and cultural settings, and sensitivity to changes in viral suppression thresholds and ART regimens (e.g. change from non-nucleoside reverse transcriptase inhibitors [NNRTIs] first line to Dolutegravir).

Future research should focus on refining and (or) updating the developed risk calculator for PPWH with new HIV diagnoses by increasing the sample size and incorporating additional relevant demographic (e.g. household income or proxy wealth index, education level, employment status, household size, travel distance to clinic), clinical (e.g. history of opportunistic infections such as Tuberculosis, pregnancy complications), and psychosocial variables (e.g. level of social support, alcohol or substance use). Prospective studies are also needed to validate both models’ effectiveness in real-world clinical settings and across diverse populations while exploring implementation strategies that are feasible and acceptable. Moreover, exploring further development using advanced machine-learning techniques could further enhance models’ predictive accuracy and improve clinical utility by reducing the number of variables and length of scales/measures required [28, 72–74].

## Conclusions

In conclusion, this study developed and validated a novel risk calculator for PPWH, which represents an advancement in the ability to predict treatment failure and disengagement from care in this population. The risk calculator for PPWH who have a known HIV diagnosis performed particularly well. This study’s innovative, multidimensional approach to identifying risk of suboptimal outcomes offers practical benefits for both clinical care and research, providing a valuable tool for early identification and intervention. Continued research and external validation efforts will be essential to optimize the model and fully realize its potential to improve health outcomes for PPWH in real-world settings.

## Electronic Supplementary Material

Below is the link to the electronic supplementary material.

Supplementary Material 1

Supplementary Material 2

## Data Availability

A request for de-identified study datasets may be made and discussed directly with the respective study principal investigators: MOTIVATE (lisa.abuogi@cuanschutz.edu or jmturan@uab.edu) and OPT4Mamas (renapatel@uabmc.edu).
