# Supplementary material for: Development and Validation of a Novel Risk Calculator to Predict Sub-optimal HIV Outcomes Among Pregnant and Postpartum Women with HIV in Kenya
Source: AIDS Behav. 2025 Jul 10;29(12):3757–68. doi: 10.1007/s10461-025-04814-8 (PMC12335848; doi:10.1007/s10461-025-04814-8)
Supplement: Supplementary file 1 — Supplementary Material 1 [file 10461_2025_4814_MOESM1_ESM.pdf]

Data Dictionary Codebook

Tatua Risk Calculator (PID: [redacted])

01/09/2025 8:09am

Instruments	
**Instrument**	**Form Name**
Risk assessment screener questions	risk_assessment_screener_questions
Patient reported questions	patient_reported_questions
Clinician data entry fields	clinician_data_entry_fields
Risk Score calculations	risk_score_calculations

Languages	
**ID**	**Display Name**
en	☑ English **(default)**
luo	☐ Luo
sw	☐ Swahili

#	Variable / Field Name	Field Label *Field Note*	Field Attributes (Field Type, Validation, Choices, Calculations, etc.)
**Instrument: Risk assessment screener questions** (risk_assessment_screener_questions)			
Active languages: None			
1	[study_id_np]	Study ID	text, Required, Identifier
2	[date_consent_np]	Date subject signed consent<br>*DD-MM-YYYY*	text (date_dmy), Required, Identifier
3	[kp_np]	Was the patient diagnosed with HIV during the current pregnancy?	dropdown, Required<br>1 Yes-New Positive<br>2 No-Known Positive
4	[dobr_np]	Date of birth	text (date_dmy), Required, Identifier
5	[age_np]	Age (years)	calc<br>Calculation: rounddown(datediff([dobr_np],[date_consent_np],'y'))
6	[risk_assessment_screener_questions_complete]	Section Header: *Form Status*<br>Complete?	dropdown<br>0 Incomplete<br>1 Unverified<br>2 Complete
**Instrument: Patient reported questions** (patient_reported_questions) Enabled as survey			
Active languages - Data Entry: en, luo, sw | Survey: en, luo, sw			
7	[language]	Please select language to complete the survey (English/ Swahili/ Luo)<br>*Select English Language / Chagua lugha ya Swahili / Yier Dholuo*	dropdown, Required<br>en English<br>sw Swahili<br>luo Luo<br>Field Annotation: @LANGUAGE-SET @LANGUAGE-CURRENT-FORM
8	[age]	Section Header: *Participant Characteristics*<br>Participant age<br>*Auto filled from records*	calc, Required<br>Calculation: [age_np]
9	[marital_status]	What is your marital status?	radio, Required<br>0 Married<br>1 Single<br>2 Living with a partner<br>3 Divorced<br>4 Widowed
10	[num_preg]	How many pregnancies have you had including this one?<br>*Select 1 for 'current pregnancy only'*	dropdown, Required<br>1 1<br>2 2<br>3 3<br>4 4<br>5 5<br>6 6<br>7 7<br>8 8<br>9 9<br>10 10<br>11 11<br>12 12<br>13 13<br>14 14<br>15 15
11	[num_births]	How many live births have you had?<br>*Select 0 for 'no live birth'*	dropdown, Required<br>0 0<br>1 1<br>2 2<br>3 3<br>4 4<br>5 5<br>6 6<br>7 7<br>8 8<br>9 9<br>10 10

			11 11<br>12 12<br>13 13<br>14 14<br>15 15
12	[stigma1]	Section Header: *In this section, I am going to ask you how you think people will relate with you given you HIV status using the following scale: strongly disagree, disagree, agree, and strongly agree*<br>I fear discrimination if i disclose my HIV positive status to others	radio (Matrix), Required<br>1 strongly disagree<br>2 disagree<br>3 agree<br>4 strongly agree
13	[stigma2]	I fear being kicked out of my community if i disclose my HIV status to others	radio (Matrix), Required<br>1 strongly disagree<br>2 disagree<br>3 agree<br>4 strongly agree
14	[stigma3]	I am afraid of violence if I disclose my HIV positive status to others	radio (Matrix), Required<br>1 strongly disagree<br>2 disagree<br>3 agree<br>4 strongly agree
15	[stigma4]	I am afraid of losing my job if I disclose my HIV status to my boss or others	radio (Matrix), Required<br>1 strongly disagree<br>2 disagree<br>3 agree<br>4 strongly agree
16	[stigma5]	I fear being kicked out of my house if I disclose my HIV positive status to others	radio (Matrix), Required<br>1 strongly disagree<br>2 disagree<br>3 agree<br>4 strongly agree
17	[liv_hiv1]	Section Header: *Now, if I may, I would like to ask you some questions about living with HIV. Many people are upset after finding out they have HIV< which is very normal and often, expected. During this next section, I am going to ask you how often in the past 12 months you felt certain ways using the following scale: never, rarely, sometimes, or often.*<br>In the last 12 months, have you felt ashamed because of your HIV status?	radio (Matrix), Required<br>1 Never<br>2 Rarely<br>3 Sometimes<br>4 Often
18	[liv hiv2]	In the last 12 months, have you felt guilty because of your HIV status	radio (Matrix), Required<br>1 Never<br>2 Rarely<br>3 Sometimes<br>4 Often
19	[liv_hiv3]	In the last 12 months, have you blamed yourself because of your HIV status	radio (Matrix), Required<br>1 Never<br>2 Rarely<br>3 Sometimes<br>4 Often
20	[liv_hiv4]	In the last 12 months, have you blamed others because of your HIV status?	radio (Matrix), Required<br>1 Never<br>2 Rarely<br>3 Sometimes<br>4 Often
21	[liv_hiv5]	In the last 12 months, have you had low self-esteem because of your HIV status?	radio (Matrix), Required<br>1 Never<br>2 Rarely<br>3 Sometimes<br>4 Often
22	[liv_hiv6]	In the last 12 months, have you felt you should be punished because of your HIV status?	radio (Matrix), Required<br>1 Never<br>2 Rarely<br>3 Sometimes<br>4 Often
23	[mps1]	Section Header: *Some women report their male partners may or may not help them with the following aspects of medical care. Please tell me since your last study visit or in the last year (if this is your first visit), if your male partner did any of the following things. These questions also have a range of answers, but I will ask them based on the following scale: all the time, most of the time, occasionally, rarely, and never.*<br>Came with you to healthcare visits? (ANC, PMTCT, or Postnatal/ Well-childcare)	radio (Matrix), Required<br>4 All the time<br>3 Most of the time<br>2 Occasionally<br>1 Rarely<br>0 Never
24	[mps2]	Encouraged you to deliver or give birth at a clinic?	radio (Matrix), Required<br>4 All the time

			3 Most of the time<br>2 Occasionally<br>1 Rarely<br>0 Never
25	[mps3]	Reminded you to take your HIV medications or if newly diagnosed with HIV, other medications, such as prenatal vitamins	radio (Matrix), Required<br>4 All the time<br>3 Most of the time<br>2 Occasionally<br>1 Rarely<br>0 Never
26	[mps4]	Reminded you to go for HIV or PMTCT care (or, if newly diagnosed, then for ANC or other medical care) ?	radio (Matrix), Required<br>4 All the time<br>3 Most of the time<br>2 Occasionally<br>1 Rarely<br>0 Never
27	[mps5]	Gave you transport money to go to the clinic or dispensary?	radio (Matrix), Required<br>4 All the time<br>3 Most of the time<br>2 Occasionally<br>1 Rarely<br>0 Never
28	[mps6]	Provided you with food or nutritional support?	radio (Matrix), Required<br>4 All the time<br>3 Most of the time<br>2 Occasionally<br>1 Rarely<br>0 Never
29	[mps7]	Reminded you to give HIV prophylaxis medication to the baby?	radio (Matrix), Required<br>4 All the time<br>3 Most of the time<br>2 Occasionally<br>1 Rarely<br>0 Never
30	[mps8]	Helped give HIV prophylaxis medication to the baby?	radio (Matrix), Required<br>4 All the time<br>3 Most of the time<br>2 Occasionally<br>1 Rarely<br>0 Never
31	[mps9]	Encouraged you to feed the baby in a certain way?	radio (Matrix), Required<br>4 All the time<br>3 Most of the time<br>2 Occasionally<br>1 Rarely<br>0 Never
32	[mps10]	Encouraged you to take the baby for HIV testing?	radio (Matrix), Required<br>4 All the time<br>3 Most of the time<br>2 Occasionally<br>1 Rarely<br>0 Never
33	[dep1]	Section Header: *Next, I would like to ask you some questions about how you have been feeling lately. It is common for some people to experience sadness or loss of interest in doing things sometimes in their lives. Over the last 2 weeks, how often have you experienced any of the following problems?*<br>Little interest or pleasure in doing things	radio (Matrix), Required<br>0 Not at all<br>1 Several days<br>2 More than half the days<br>3 Nearly every day
34	[dep2]	Feeling down, depressed, or hopeless	radio (Matrix), Required<br>0 Not at all<br>1 Several days<br>2 More than half the days<br>3 Nearly every day
35	[dep3]	Trouble falling or staying asleep, or sleeping too much	radio (Matrix), Required<br>0 Not at all<br>1 Several days<br>2 More than half the days<br>3 Nearly every day
36	[dep4]	Feeling tired or having little energy	radio (Matrix), Required

			0 Not at all<br>1 Several days<br>2 More than half the days<br>3 Nearly every day
37	[dep5]	Poor appetite or overeating	radio (Matrix), Required<br>0 Not at all<br>1 Several days<br>2 More than half the days<br>3 Nearly every day
38	[dep6]	Feeling bad about yourself - or that you are a failure or have let yourself or your family down	radio (Matrix), Required<br>0 Not at all<br>1 Several days<br>2 More than half the days<br>3 Nearly every day
39	[dep7]	Trouble concentrating on things, such as reading the newspaper or watching television	radio (Matrix), Required<br>0 Not at all<br>1 Several days<br>2 More than half the days<br>3 Nearly every day
40	[dep8]	Moving or speaking so slowly that other people could have noticed? Or the opposite - being so fidgety or restless that you have been moving around a lot more than usual	radio (Matrix), Required<br>0 Not at all<br>1 Several days<br>2 More than half the days<br>3 Nearly every day
41	[ipv1]	Section Header: *The next set of questions is about things that happen to many couples. Please indicate if any of the following things happened to you in the past 6 months with a current or former partner. When two people are in a relationship, they usually share both good and bad moments. I would like to ask you some questions about your current and past relationships and how your partner treats (treated) you. This may be a sensitive section for you. Please feel free to take some time before answering questions if you need to do so. I would again like to assure you that your answers will be kept secret from others including your partner. If, however, we find that your safety is at risk, we may need to seek additional help to ensure your safety. In the unlikely event, that someone interrupts us, I will change the topic of conversation. May I continue?*<br>My partner slapped me or threw something at me that could hurt me	radio (Matrix), Required<br>0 never<br>1 Not in the past 6 months but happened before<br>2 Once in the past 6 months<br>3 Twice in the past 6 months<br>4 Three, or more times in the past 6 months
42	[ipv2]	My partner pushed me or shoved me	radio (Matrix), Required<br>0 never<br>1 Not in the past 6 months but happened before<br>2 Once in the past 6 months<br>3 Twice in the past 6 months<br>4 Three, or more times in the past 6 months
43	[ipv3]	My partner hit me with his !st or with something else that could hurt me	radio (Matrix), Required<br>0 never<br>1 Not in the past 6 months but happened before<br>2 Once in the past 6 months<br>3 Twice in the past 6 months<br>4 Three, or more times in the past 6 months
44	[ipv4]	My partner kicked me, dragged me or beat me up	radio (Matrix), Required<br>0 never<br>1 Not in the past 6 months but happened before<br>2 Once in the past 6 months<br>3 Twice in the past 6 months<br>4 Three, or more times in the past 6 months
45	[ipv5]	My partner choked or burnt me on purpose	radio (Matrix), Required<br>0 never<br>1 Not in the past 6 months but happened before<br>2 Once in the past 6 months<br>3 Twice in the past 6 months<br>4 Three, or more times in the past 6 months
46	[ipv6]	My partner threatened to use or actually used a gun, knife or other weapon against me	radio (Matrix), Required<br>0 never<br>1 Not in the past 6 months but happened before<br>2 Once in the past 6 months<br>3 Twice in the past 6 months<br>4 Three, or more times in the past 6 months
47	[food_access]	Section Header: *Food Insecurity Question*<br>In the past 4 weeks, how often did it happen that there was no food to eat of any kind in your house, because of lack of resources to get food?	radio, Required<br>0 Never<br>1 Rarely<br>2 Sometimes<br>3 Often
48	[told_status]	Section Header: *Disclosure of HIV status*<br>Have you told anyone about your HIV status?	dropdown, Required<br>0 No<br>1 Yes

49	[told_status_pat]	Have you told your male partner about your HIV status?<br>*Select 'N/A' if not applicable*	dropdown, Required<br>0 No<br>1 Yes<br>2 N/A
50	[patient_reported_questions_complete]	Section Header: *Form Status*<br>Complete?	dropdown<br>0 Incomplete<br>1 Unverified<br>2 Complete
**Instrument: Clinician data entry fields (clinician_data_entry_fields)**			
Active languages: None			
51	[gest_age]	Gestational age (weeks) at first prenatal visit<br>*Check gestational age from ANC register*	text (number, Min: 0, Max: 42), Required
52	[hist_missed_visits]<br>Show the field ONLY if:<br>[kp_np] = '2'	History of missed clinic visits (>14 days continuously) in the past 12 months?<br>*Check for any scheduled visit date that has been missed by more than 14 days by comparing it with actual visit date*	dropdown, Required<br>0 No<br>1 Yes
53	[base_vl]<br>Show the field ONLY if:<br>[kp_np] = '2'	Baseline viral load [nearest to study enrolment within +/- 3 months window]<br>*Enter '-999' if missing baseline viral load*	text (integer), Required
54	[hist_elev_vl]<br>Show the field ONLY if:<br>[kp_np] = '2'	History of elevated viral load >=400 copies/ml in the past 12 months	dropdown, Required<br>0 No<br>1 Yes
55	[art_reg]<br>Show the field ONLY if:<br>[kp_np] = '2'	ART Regimen<br>*Choose regimen from the drop down*	dropdown, Required<br>1 TDF/3TC/NVP<br>2 TDF/3TC/EFV<br>3 AZT/3TC/NVP<br>4 AZT/3TC/EFV<br>5 D4T/3TC/NVP<br>6 D4T/3TC/EFV<br>7 TDF/3TC/AZT<br>8 AZT/3TC/DTG<br>9 TDF/3TC/DTG<br>10 AZT/3TC/LPV/r<br>11 AZT/3TC/ATV/r<br>12 TDF/3TC/ATV/r<br>13 TDF/3TC/LPV/r<br>14 B/F/TAF<br>15 ABC/3TC/DTG<br>16 ABC/3TC/EFV<br>17 ABC/3TC/NVP<br>18 AZT/3TC/LPV/r<br>19 AZT/3TC/ATV/r<br>20 TDF/3TC/LPV/r<br>21 TDF/3TC/ATV/r<br>22 D4T/3TC/LPV/r<br>23 AZT/TDF/3TC/LPV/r<br>24 ETR/RAL/DRV/RTV<br>25 ETR/TDF/3TC/LPV/r<br>26 ABC/3TC/LPV/r<br>27 ABC/3TC/ATV/r<br>28 AZT/3TC/DTG<br>29 TDF/3TC/DTG<br>30 ABC/3TC/DTG<br>31 AZT/3TC/LPV/r<br>32 AZT/3TC/ATV/r<br>33 AZT/3TC/DTG<br>34 TDF/3TC/LPV/r<br>35 TDF/3TC/DTG<br>36 TDF/3TC/ATV/r<br>37 ABC/3TC/LPV/r<br>38 ABC/3TC/ATV/r<br>39 ABC/3TC/DTG<br>40 TDF/3TC/DTG/DRV/RTV<br>41 TDF/3TC/RAL/DRV/RTV<br>42 RAL/BTC/DRV/RTV<br>43 RAL/3TC/DRV/RTV/AZT<br>44 RAL/BTC/DRV/RTV/TDF<br>45 ETV/3TC/DRV/RTV<br>46 TDF/3TC/DTG/DRV/r<br>47 TDF/3TC/RAL/DRV/r

			48 TDF/3TC/DTG/EFV/DRV/r<br>49 TDF/BC/DTC<br>50 TDF/3TC/DTG/ATV/r<br>51 TDF/3TC/DTG/ETV/DRV/r<br>52 ABC/ST/DTC/DRV/r
56	[badh]<br>Show the field ONLY if:<br>[kp_np] = '2'	Baseline Adherence (Poor, Good/Excellent, or Unknown)<br>*Select one from the list*	dropdown, Required<br>1 Good/Excellent<br>2 Poor<br>3 Unknown
57	[clinician_data_entry_fields_complete]	Section Header: *Form Status*<br>Complete?	dropdown<br>0 Incomplete<br>1 Unverified<br>2 Complete
**Instrument: Risk Score calculations (risk_score_calculations)**			
Active languages: None			
58	[agele26_np]	Section Header: *Demographics Items*<br>Young age [< 26 years] (Yes/No)	calc<br>Calculation: if([age_np] < 26,1,0)
59	[notmarried_np]	Not Married (Yes/No)	calc<br>Calculation: if([marital_status] >=1,1,0)
60	[nullip_np]	Nulliparous Parity (Yes/No)	calc<br>Calculation: if([num_births] =0,1,0)
61	[gestage_np]	Section Header: *Clinical Items*<br>Gestational age first ANC (weeks)	calc<br>Calculation: if([gest_age] > 26,1,0)
62	[gestagele26_np]	Gestational age first ANC [>26 weeks] (Yes/No)	calc<br>Calculation: if([gestage_np] >26,1,0)
63	[bvl_g1000]	Baseline Viral Load [+/- 3 months] >=1000 copies (Yes/No or Unknown)	calc<br>Calculation: if([base_vl] >=1,1,0)
64	[bvl_unk]	Baseline Viral Load Unknown (Yes/No)	calc<br>Calculation: if([base_vl] =-999,1,0)
65	[hist_elev_vlnp]	History of elevated viral load >=400 copies/ml in the past 12 months (Yes/No)	calc<br>Calculation: [hist_elev_vl]
66	[badh_good]	Baseline Adherence [Good] (Yes/No)	calc<br>Calculation: if([badh] = 1,1,0)
67	[badh_unk]	Baseline Adherence [Unknown] (Yes/No)	calc<br>Calculation: if([badh] = 3,1,0)
68	[art_regnp]	ART Regimen [Second- or third-line] (Yes/No)	calc<br>Calculation: if([art_reg] >17,1,0)
69	[int_part_vio_np]	Section Header: *Psychosocial Items*<br>Intimate Partner Violence (Yes/No)	calc<br>Calculation: if([ipv1] >0 or [ipv2] >0 or [ipv3] >0 or [ipv4] >0 or [ipv5] >0 or [ipv6] >0 , 1, 0)
70	[ant_hiv_stig_np]	Anticipated HIV stigma (Yes/No)	calc<br>Calculation: if([stigma1] >2 or [stigma2]>2 or [stigma3]>2 or [stigma4]>2 or [stigma5]>2, 1, 0)
71	[int_hiv_stig_np]	Internalized HIV stigma (Yes/No)	calc<br>Calculation: if([liv_hiv1] >1 or [liv_hiv2] >1 or [liv_hiv3] >1 or [liv_hiv4] >1 or [liv_hiv5] >1 or [liv_hiv6] >1,1,0)
72	[malept_sup_np]	Male Partner Support [Average Score < 3] (Yes/No)	calc<br>Calculation: if(mean([mps1],[mps2],[mps3],[mps4],[mps5],[mps6],[mps7],[mps8],[mps9],[mps10]) < 3,1,0)
73	[major_dep]	Major Depression PHQ-9 [Total Score >9] (Yes/No)	calc<br>Calculation: if(([dep1]+[dep2]+[dep3]+[dep4]+[dep5]+[dep6]+[dep7]+[dep8] >9), 1,0)
74	[nondisc_pat]	Non disclosure of HIV status to male partner (Yes/No)	calc<br>Calculation: if([told_status_pat]=0,1,0)
75	[food_insec_np]	Food Insecurity (Yes/No)	calc<br>Calculation: if([food_access] >0, 1, 0)
76	[logodds_np]	Section Header: *Risk Score Calculation and Risk Group*<br>Total Log Odds	calc<br>Calculation: if(([kp_np] = '2') , (-2.1117251 - (0.0542308*[agele26_np]) + (0.0466795*[notmarried_np]) + (0.633511*[bvl_g1000]) + (0.6370091*[bvl_unk]) + (0.5057918*[hist_elev_vlnp]) + (3.2216449*[hist_missed_visits]) - (0.4935859*[int_part_vio_np]) + (0.5759957*[ant_hiv_stig_np]) - (0.4696528*[int_hiv_stig_np]) - (0.0394667*[major_dep]) + (0.7031839*[malept_sup_np]) + (0.2452464*[nondisc_pat]) + (0.2523488*[art_regnp]) - (0.3161543*[badh_unk]) + (0.1889777*[badh_good]) - (0.2478611*[food_insec_np])) , (0.2176235 + (0.2838567*[nullip_np]) + (0.6523616*[ant_hiv_stig_np]) + (0.3518472*[malept_sup_np]) - (0.1366500*[agele26_np]) - (0.3106959*[notmarried_np]) - (0.0800222*[gestage_np]) - (0.148019*[int_part_vio_np]) - (0.5977594*[int_hiv_stig_np]) - (0.3967156*[food_insec_np])))
77	[risk_np_2]	Risk Score [Range: 0-1] (3 decimal places)	calc<br>Calculation: if([kp_np]='1', exponential((0.2176235 - 0.1366500*[agele26_np] - 0.3106959*[notmarried_np] + 0.2838567*[nullip_np] - 0.0800222*[gestage_np] - 0.148019*[int_part_vio_np] + 0.6523616*[ant_hiv_stig_np] - 0.5977594*[int_hiv_stig_np] + 0.3518472*[malept_sup_np] - 0.3967156*[food_insec_np]))/(1+exponential((0.2176235 - 0.1366500*[agele26_np] - 0.3106959*[notmarried_np] + 0.2838567*[nullip_np] - 0.0800222*[gestage_np] - 0.148019*[int_part_vio_np] + 0.6523616*[ant_hiv_stig_np] - 0.5977594*[int_hiv_stig_np] + 0.3518472*[malept_sup_np] - 0.3967156*[food_insec_np]))), exponential((-2.1117251 - 0.0542308*[agele26_np] + 0.0466795*[notmarried_np] + 0.633511*[bvl_g1000] + 0.6370091*[bvl_unk] + 0.5057918*[hist_elev_vlnp] + 3.2216449*[hist_missed_visits] - 0.4935859*[int_part_vio_np] + 0.5759957*[ant_hiv_stig_np] -

			0.4696528*[int_hiv_stig_np] - 0.0394667*[major_dep] + 0.7031839*[malept_sup_np] + 0.2452464*[nondisc_pat] + 0.2523488*[art_regnp] - 0 3161543*[badh unk] + 0 1889777*[badh good] 0 2478611*[food_insec_np]))/(1 + exponential((-2.1117251 - 0.0542308*[agele26_np] + 0.0466795*[notmarried_np] + 0.633511*[bvl_g1000] + 0.6370091*[bvl_unk] + 0.5057918*[hist_elev_vlnp] + 3.2216449*[hist_missed_visits] - 0.4935859*[int_part_vio_np] + 0.5759957*[ant_hiv_stig_np] - 0.4696528*[int_hiv_stig_np] - 0.0394667*[major_dep] + 0.7031839*[malept_sup_np] + 0.2452464*[nondisc_pat] + 0.2523488*[art_regnp] - 0.3161543*[badh_unk] + 0.1889777*[badh_good] - 0.2478611*[food_insec_np]))))
78	[risk_grp_np]	Risk group<br>*1- Low Risk, 2 - Medium Risk, 3 - High Risk*	text<br>Field Annotation: @CALCTEXT(if([kp_np]='2' and [risk_np_2] <= 0.157,'1 - Low Risk', if([kp_np]='2' and [risk_np_2] > 0.157 and [risk_np_2] <= 0 702, '2 Medium Risk', if([kp np] '2' and [risk np 2] > 0 702, '3 High Risk', if([kp_np]='1' and [risk_np_2] <= 0.405, '1 - Low Risk', if([kp_np]='1' and [risk_np_2] > 0.405 and [risk_np_2] <= 0.546, '2 - Medium Risk', if([kp_np]='1' and [risk_np_2] > 0.546, '3 - High Risk',''))))))
79	[risk_score_calculations_complete]	Section Header: *Form Status*<br>Complete?	dropdown<br>0 Incomplete<br>1 Unverified<br>2 Complete
