# Supplementary material for: Development and Validation of a Novel Risk Calculator to Predict Sub-optimal HIV Outcomes Among Pregnant and Postpartum Women with HIV in Kenya
Source: AIDS Behav. 2025 Jul 10;29(12):3757–68. doi: 10.1007/s10461-025-04814-8 (PMC12335848; doi:10.1007/s10461-025-04814-8)
Supplement: Supplementary file 2 — Supplementary Material 2 [file 10461_2025_4814_MOESM2_ESM.pdf]

REDCap

Tatua Risk Calculator
Study ID [redacted]

Save & Exit Form
Save & Stay
-- Cancel --

Risk Score calculations

**Study ID**	[redacted]
**Demographics Items**	
**Young age [< 26 years] (Yes/No)**	1
**Not Married (Yes/No)**	1
**Nulliparous Parity (Yes/No)**	1
**Clinical Items**	
**Gestational age first ANC (weeks)**	1
**Gestational age first ANC [>26 weeks] (Yes/No)**	0
**Baseline Viral Load [+/- 3 months] >=1000 copies (Yes/No or Unknown)**	0
**Baseline Viral Load Unknown (Yes/No)**	0
**History of elevated viral load >=400 copies/ml in the past 12 months (Yes/No)**	0
**Baseline Adherence [Good] (Yes/No)**	1
**Baseline Adherence [Unknown] (Yes/No)**	0
**ART Regimen [Second- or third-line] (Yes/No)**	0
**Psychosocial Items**	
**Intimate Partner Violence (Yes/No)**	0
**Anticipated HIV stigma (Yes/No)**	1
**Internalized HIV stigma (Yes/No)**	1
**Male Partner Support [Average Score < 3] (Yes/No)**	1
**Major Depression PHQ-9 [Total Score >9] (Yes/No)**	0
**Non disclosure of HIV status to male partner (Yes/No)**	0
**Food Insecurity (Yes/No)**	1
**Risk Score Calculation and Risk Group**	
**Total Log Odds**	-1.368633
**Risk Score [Range: 0-1] (3 decimal places)**	0.202840795632206
**Risk group**	2 - Medium Risk<br>1- Low Risk, 2 - Medium Risk, 3 - High Risk
**Form Status**	
**Complete?**	Complete
